# Minimally Invasive Vitreoretinal Surgery: Is Sutureless Vitrectomy the Future of Vitreoretinal Surgery?

**Published:** 2011-04

## Introduction

Pars plana vitrectomy (PPV) was introduced almost 40 years ago.^1^ In the 1980s and 1990s, three-port PPV with 20-gauge (G) instruments was the norm. In 2002, 25-gauge transconjunctival sutureless vitrectomy (TSV) was introduced.^2^,^3^ This system permits three-port PPV using microcannulas, trocars, and 25-G instrumentation without requiring sutures to close the sclerotomies. Subsequently, a similar technique but with 23-G instruments was developed.^4^ Currently, 25- and 23-gauge systems constitute the two most popular TSV techniques.

Herein, we review the advantages and disadvantages of small-gauge vitreous surgery.

## Instrumentation

**Figure 1:**
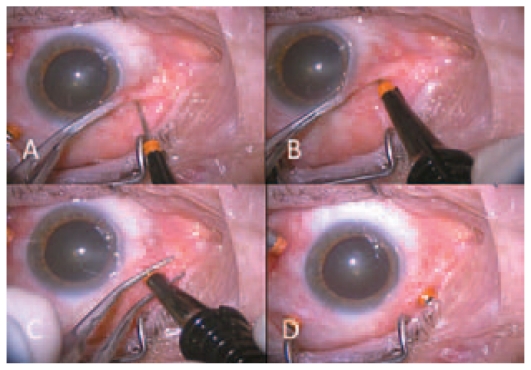
Insertion of 23-gauge trocars and microcannulas. The microcannulas are inserted through the conjunctiva into the eye by means of a trocar. Insertion is accomplished by first displacing the conjunctiva laterally by approximately 2 mm. An initially oblique, then perpendicular tunnel is made parallel to the limbus through the conjunctiva and sclera, thus creating a self-sealing wound.

TSV consists of a 23-G or 25-G microcannular system and a wide array of vitreoretinal instruments specifically designed for this operating system. The microcannula consists of a thin-walled tube, 4 mm in length. A collar is present at the extraocular portion, which can be grasped with a forceps to manipulate the microcannula. The insertion trocar has a sharp tip that forms a continuous bevel with the microcannula, allowing easy entry through the conjunctiva into the eye (Fig. 1). The 25-G infusion cannula consists of a small tube that fits neatly and can be directly inserted into the cannula in the inferotemporal quadrant. A wide array of vitreoretinal microsurgical instruments complying with 25-G standards has been designed. These include vitreous cutters, illumination probes, intraocular forceps, microvitreoretinal blades, tissue manipulators, aspirating picks, aspirators, soft-tip cannulas, curved scissors, extendable curved picks, intraocular laser probes, and diathermy probes.

## Surgical Technique

Small gauge vitrectomy is usually performed with the patient under local anesthesia. General anesthesia is only performed in selected cases (children or uncooperative adults). After appropriate anesthesia, the operative field is prepared using antiseptic solutions. Preoperatively, the eyelash margins are scrubbed with povidone-iodine solution. The microcannulas are inserted through the conjunctiva into the eye by means of a trocar. Insertion is accomplished by first displacing the conjunctiva laterally by approximately 2 mm. An initially oblique, then perpendicular tunnel is made parallel to the limbus through the conjunctiva and sclera creating a self-sealing wound (Fig. 1). After insertion of the first microcannula, the intraocular portion of the infusion cannula is directly inserted into the external opening of the microcannula.^5^,^6^ Once the intraocular position of the infusion cannula is verified, infusion is opened and the other two microcannulas are inserted in the superotemporal and superonasal quadrants for three-port PPV. At the completion of surgery, the microcannulas are simply removed by grasping the collar and withdrawing, along with assessment of intraocular pressure (IOP) and wound sites for possible leaks.

**Figure 2:**
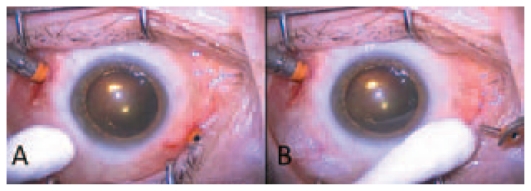
After withdrawal of the cannula, a cotton tip applicator can be used to misalign the outer and inner aspects of the sclerotomies thereby reducing the risk of leakage. subsided due to recent studies revealing a low incidence of endophthalmitis in a large series of patients undergoing small-gauge sutureless surgery.^8^

The 23-G system is a variation of the 25-G TSV system. 23-G vitreous cutters have been improved by placing the cutter opening nearer to the end of the probe allowing a closer vitreous shave. This increases safety near the retina. At the end of vitrectomy, adequate gas/air tamponade must be performed which avoids significant postoperative leakage in most cases. However, in some cases leakage may occur and the sclerotomy site should be closed with a single 7–0 or 8–0 vycril suture. In addition, sclerotomy sites are to be closed if silicone oil is used. The microcannulas can be simply removed by grabbing the external collar with a forceps at the end of the procedure. The last microcannula to be removed should be the one with the infusion line (Fig. 2). Postoperative subconjunctival injection of antibiotic and steroid solutions should be administered as in standard vitrectomy. Endophthalmitis is extremely uncommon following vitreous surgery, but there is a theoretical concern that 25-G sutureless surgery may pose an added risk.^7^ However, these concerns have

## Advantages of Small-gauge Vitrectomy

**Figure 3:**
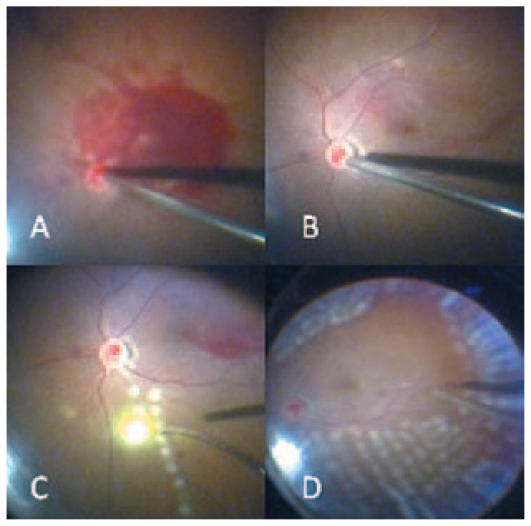
Intraoperative photographs of a diabetic eye with vitreous and preretinal hemorrhages managed with 23-gauge instrumentation.

**Figure 4:**
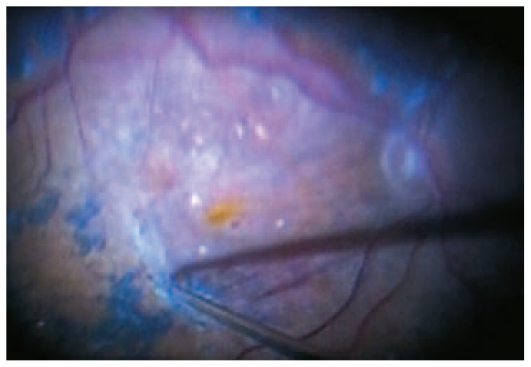
Intraoperative photograph of macular epiretinal membrane treated with 23-gauge instrumentation.

In general, TSV seems to be particularly advantageous for procedures that do not require extensive intraocular tissue dissection or manipulation. Experience has shown that 25-gauge surgery is ideal for vitreous and preretinal hemorrhages in proliferative diabetic retinopathy (Fig. 3), rhegmatogenous retinal detachment, proliferative vitreoretinopathy (PVR), giant retinal breaks, and cases in which vitrectomy and phacoemulsification are combined with intraocular lens (IOL) implantation. It is also applicable for diabetic traction retinal detachment with moderate amounts of epiretinal membranes (ERMs), and idiopathic ERMs as well (Fig. 4). However, if scleral buckling or silicone oil tamponade is anticipated, standard 20-G vitrectomy is preferred as its full capability may be required in those cases. Even in complex cases where one needs a variety of scissors and forceps and/or the injection of silicone oil, 25-G or 23-G sclerotomies can be used for the infusion and illumination probes, and a 20-G sclerotomy can be performed for the instruments and the injection and removal of silicone oil. This enables the surgeon to use 20-G instruments and reduce the cost of replacing all devices.^9^ An alternative is to use 1000 centistoke silicone oil with 23-G instrumentation, therefore even complex cases can be resolved with small-gauge surgery.

**Figure 5:**
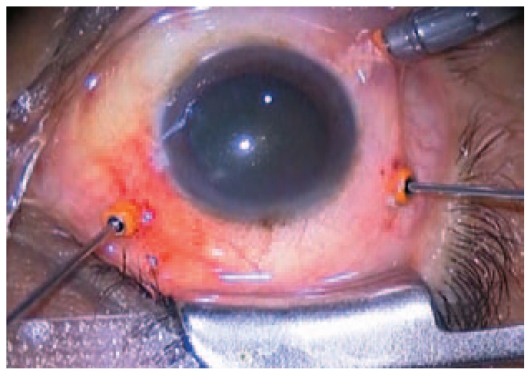
The microcannulas are inserted through the conjunctiva into the eye and 23-gauge instruments are in place at the sclerotomy sites.

Another advantage of TSV becomes apparent in pediatric cases. Typically, newborn and premature eyes are significantly smaller than adult eyes and the use of standard vitreoretinal instruments may be technically difficult.^10^ With TSV, the intraocular instruments are more compatible with the smaller pediatric eyes and are efficient in selected cases of persistent fetal vasculature, retinopathy of prematurity, uveitis, and some cases of uncomplicated tractional or rhegmatogenous retinal detachments. In addition, TSV offers benefits in certain vitreoretinal cases because it is transconjunctival. TSV-based surgery has the potential to shorten operative time for a variety of procedures, and reduce postoperative inflammation at the sclerotomy sites (Fig. 5), thus reducing patient discomfort and hastening postoperative recovery. It also avoids induced astigmatism, allowing more rapid visual recovery.^11^

## Disadvantages of Small-gauge Vitrectomy

One disadvantage of this system is the learning curve required to achieve maximum efficiency. However, this curve is short enough for the adaptable surgeon.

Due to its smaller fiberoptic size, illumination is also reduced with 25-gauge surgery. However, current systems provide adequate illumination in most cases. A noticeable difference of 25-G instruments is their marked flexibility.

There are some potential complications specifically related to the 25-gauge system, the most obvious being hypotony and a higher incidence of endophthalmitis.^7^ The risk of these complications can be reduced by creating a tunnel or angular incision in a different plane relative to the conjunctiva, and performing fluid-air exchange at the end of the surgery. It is important to note that hypotony is more common in previously vitrectomized eyes. As mentioned, these concerns have been diminished as a result of recent studies demonstrating a low incidence of endophthalmitis in large series of patients undergoing TSV.^8^ In terms of prophylaxis, all patients undergoing 25-gauge vitrectomy should have standard and meticulous preparation with povidone-iodine, as well as postoperative injection of subconjunctival antibiotics.

For a surgeon used to performing 20-G vitrectomy, transition to 23-G is easier than 25-G. With the 23-G system, rigidity, flow and aspiration of the vitreous cutter are similar to the 20-G system, and lighting is comparable. The instruments have stiffness similar to 20-G. However the sclerotomies must be precisely formed, with tunnel or angular incisions to reduce complications.

## Recent Advances in 23- and 25-gauge Surgery: Overcoming the Disadvantages

### Instrument Rigidity

The lower rigidity of instruments is a problem with 25-G, these instruments are more pliable and more damageable, furthermore manipulation of the globe can become cumbersome. This is not an issue with 23-G, as rigidity is similar to 20-G (Fig. 5). Several companies are producing more rigid 25-G instruments (including the new Alcon Constellation system; Alcon Laboratories, Fort Worth, TX, USA) or are reducing instrument length to achieve the same purpose.

### Instrument Availability

Initially, available instruments were limited to small-gauge forceps, however nowadays a full armamentarium of instruments is available in small-gauge, including extrusion cannulas for silicone oil injection and removal, scissors, dual-bore cannulas for perfluorocarbon injection, diathermy probes, multidirectional laser probes, chandeliers, and 40-G cannulas for subretinal injections. In essence, at present, the same range of instruments used in 20-G, is available in 23- and 25-G, with the exception of a fragmatome. Nevertheless, several companies are working on developing a 23-G fragmatome to address dislocated nuclei, and DORC (Zuidland, The Netherlands) has recently released the 23-gauge Rayes Fragmentation Needle.

### Illumination

Since the number of light fibers is reduced, particularly with 25-G, brighter light sources are needed, such as Photon (Synergetics Inc., O’Fallon, MO, USA) and Xenon (Alcon Laboratories, Fort Worth, TX, USA). The new Alcon Constellation system has much brighter light than the actual Xenon. Bausch & Lomb (Rochester, NY, USA) uses the Photon. With these recent modifications, illumination is not much of an issue anymore.

### Cutting Efficiency

Slow vitreous removal is a potential problem with 25-G, this issue has been addressed in the new Alcon Constellation machine. The new 25-G probe has a bigger opening and a longer duty cycle (the amount of time the port is open); these alterations allow an increased aspiration rate while maintaining high-speed cutting rates. The 23-G system will benefit from the same duty cycle improvement, but the probe is different and does not have a spring mechanism, so it can stay open longer during each cut, allowing greater aspiration. Thus the rapidity of vitreous removal with 23- and 25-G will be markedly improved. Cutting rates of up to 5000 cuts/min are available, allowing for shaving of the vitreous base and safer vitrectomy, even in detached retinas.

### Wound Architecture

Wound architecture is the most important aspect of TSV; complications such as endophthalmitis and retinal breaks are associated with badly fashioned wounds. Initially, wounds for 25-G vitrectomy were made by direct entry, this could have been the cause of hypotony and increased endophthalmitis rates among other complications. Displacement of the conjunctiva and a two-plane wound with fluid-air exchange at the end of the procedure, reduce wound leaks and decrease the risk of endophthalmitis and hypotony. The DORC and new Alcon 23-G TSV systems have a flat blade trocar system which produces a slit wound that closes better than the actual chevron wound made by the round trocar blade system. Wound construction in our view is the most important aspect of TSV and the key point in the learning curve. One should always keep in mind that if any doubt exists regarding leakage, the surgeon should suture the sclerotomy site. Our threshold for tends to be lower in complicated cases where silicone oil needs to be used.

### Surgical Outcomes

Other benefits of TSV include astigmatism-neutral surgery, shortened operative time, less inflammation and reduced patient discomfort. We truly believe that the reportedly increased incidence of endophthalmitis is technique-dependent. With adequate preoperative povidone-iodine preparation, good wound construction in two planes, a partial or total fluid-air exchange at the end of the procedure, and subconjunctival antibiotics, the risk of hypotony and endophthalmitis can be reduced.

### Learning Curve

The learning curve is mainly due to wound construction and for 25-G, the current lack of rigidity. The TSV 23-G system has less of a learning curve, as the instruments have similar rigidity as 20-G. The 23-G system has been more user-friendly due to the illumination, rigidity, and increased flow, which are all similar to 20-G. With improvements in 25-G systems, at least with the Constellation system, 25-G surgery will feel more like 23-G, as rigidity, aspiration, and illumination are increased significantly.

## Summary

Cataract surgery was revolutionized by the introduction of phacoemulsification and foldable IOLs. This enabled a reduction in the size of the incision and avoided the necessity of using sutures. This transition shortened operative time, reduced complications, and increased patient satisfaction and comfort.

The same transition is occurring in vitreous surgery. Both vitrectomy techniques, either 25-G or 23-G, have been improving with time, experience, and the introduction of better instruments. Surgical indications are expanding with experience and the availability of new technology. Performing minimally invasive surgery has many advantages for both the surgeon and the patient. The reduced surgical time improves efficiency and also reduces complications and surgical trauma. Postoperative recovery is more rapid, since there is less inflammation. Technological developments, more efficient vitreous cutters, and a variety of 23-G and 25-G instruments have made small-gauge vitrectomy the gold standard and it is here to stay.
