# Supplementary material for: Minimally Invasive Vitreoretinal Surgery: Is Sutureless Vitrectomy the Future of Vitreoretinal Surgery?
Source: J Ophthalmic Vis Res. 2011 Apr;6(2):136–44. (PMC3306080)
Supplement: Related texts

# Other: Con

**Published:** 2011-04

**Authors:** Touka Banaee

**Affiliations:** Eye Research Center, Mashhad University of Medical Sciences, Mashhad, Iran

The large leap in surgery during the past few decades has been its progress towards becoming more minimal and less destructive. Laparoscopic surgery is a prototype of this change. This concept has also provided immense changes in ophthalmic surgical techniques: transition from intracapsular to extracapsular cataract surgery with implantation of IOLs, followed by less invasive phacoemulsification with foldable IOLs, and finally, minimally invasive cataract surgery.

But what about vitreoretinal procedures? Have these operations also moved in same direction of becoming less invasive and more minimal? With no doubt, the answer is yes. The change in buckling techniques from implants to explants, the trend to use segmental buckles instead of encircling elements, and the use of pneumatic retinopexy or vitrectomy instead of scleral buckling are all signs of attempts to do less extensive, but more effective procedures. One of the greatest changes in this field during the last two decades has been the introduction of minimally invasive vitreoretinal surgery (MIVS), which by using 23- or 25-gauge instruments, allows a sutureless procedure.^1^,^2^

Although most vitrectomy cases are suitable candidates for sutureless vitrectomy, not all patients can be handled “sutureless”. Examples include extraction of a large intraocular foreign body in a case with a clear lens, and eyes requiring scleral buckling in addition to vitrectomy. Clearly, these constitute only a minority of current vitrectomy cases. There are other situations in which the use of sutureless techniques with its small-gauge instruments still poses limitations, such as the absence of a full range of multifunctional instruments in small-gauge for complicated retinal detachments and diabetic retinopathy, flexibility of the instruments when maneuvers in the retinal periphery become necessary, inability to overcome dense thick membranes with small-gauge instruments, and the injection and removal of silicone oil, especially of high viscosity. In most of these situations, the case cannot be managed with self-sealing sclerotomies.

I am aware of the fact that the mentioned limitations are being solved by technological advances and will no longer be a problem in the near future. But let’s look at the question from another perspective.

The question whether sutureless vitrectomy is the future of vitreoretinal surgery is similar to asking whether phacoemulsification is the future of cataract surgery. I think almost everyone would agree on a negative answer. Why? Because we already have it and use it regularly. It is not the “future” of cataract surgery, it is the current state. Although phacoemulsification has been a large progress towards minimization of cataract surgery, everybody knows that phacoemulsification is still not the best minimal surgery and ophthalmologists are looking forward to enzyme-assisted phacolysis along with the injection of liquid IOLs as a realistic goal. The same also applies to sutureless vitrectomy techniques. These techniques present a state of the art procedure, and will certainly be a the predominant method for the next few years, but they are not the “future” of vitreoretinal surgery, just as phacoemulsification is not the future of cataract surgery. In my opinion, the future is in the hands of injections, along with preventive and personalized ophthalmology.

We now know that the vitreous is not only a clear substance for filling a cavity; it plays many physiological roles, the most clearly known is oxygen absorption and prevention of oxidative damage to intraocular structures.^3^ Eyes undergoing vitrectomy are prone to the development of nuclear sclerotic cataracts and open angle glaucoma, probably due to augmented oxidative damage.^4^ Therefore removal of the vitreous may not always be desirable. If a treatment or preventive measure becomes available that by producing syneresis preserves gel vitreous, it will surely be a better option than surgery for many of current indications for vitrectomy.

A common saying in medicine is “prevention beats treatment”. Surely, progress in medical knowledge and resources will move the medical community more and more towards “preventive medicine”. To achieve prevention, one must first recognize those at risk, and personalized medicine will prove a powerful tool in this regard. In the personalized medical strategy, which is the beginning of a new era in all medical fields, information based on evaluation of genetic variations and expression profiles, proteins, and markers, is used to deliver targeted treatment or enforce prevention. Recognizing patients at risk for developing various diseases and obtaining knowledge on how the disease and the response to treatment is expressed or modified by the genetic background of the patient, are turning this idea into reality.^5^,^6^ It is conceivable that in the future, we may be able to recognize genes causing abnormal vitreoretinal adhesions in patients developing vitreoretinal interface problems, macular holes, and retinal breaks after posterior vitreous detachment (PVD). We may even become able to recognize diabetics at risk of developing proliferative disease by evaluating their genetic background, and apply preventive measures in susceptible cases.

Chemical vitreolysis has drawn the attention of vitreoretinal surgeons for many years.^7^ Many agents have been examined in this regard: hyaluronidase^8^, autologous plasmin^9^,^10^, tissue plasminogen activator^11^,^12^, various enzymes^13^,^14^, and more recently, microplasmin^15^. But why chemical vitreolysis? Because injection is easier and less time-consuming than an operation, it creates a smoother surface with a lower risk of re-proliferation, and is less traumatic to the retina. Microplasmin is a synthetic recombinant form of truncated plasmin which cleaves fibronectin and laminin molecules at the vitreoretinal interface.^16^ In a randomized clinical trial, a single injection of microplasmin in eyes with vitreomacular traction, induced PVD in 44% of eyes 28 days after the injection, and in 58% after multiple injections.^15^

With the discovery of additional targeted molecules, chemical vitreolysis will surely open its way as a safe and effective procedure. Targeted treatment to weaken the vitreoretinal junction without causing separation from the retina may become possible in the near future. This may have merit for prophylaxis against retinal break formation by PVD in susceptible eyes. Prevention of retinal break formation would circumvent one of the major indications for vitrectomy, i.e., retinal detachment and PVR. The same goal may prove desirable in those predisposed to macular hole formation and other vitreoretinal interface abnormalities. In other situations, such as diabetic retinopathy, it may be preferred to induce complete PVD in order to prevent new vessel growth into the vitreous cortex before development of proliferative retinopathy. By preventing proliferative diabetic retinopathy and its complications, one of the most common indications for vitrectomy will be avoided.

It seems that personalized medicine, along with advances in the field of chemical vitreolysis, will help ophthalmologists prevent many of the current indications for vitrectomy in the future. I admit that in spite of all the mentioned strategies, the need for vitrectomy surgery will remain for some cases.

Injections can also be used for other purposes, such as gene therapy, stem cell therapy, and the delivery of slow-release drug implants. These strategies may pave the road for treatment or prevention of many currently untreatable diseases, such as retinal degenerations and end-stage glaucoma.

With the preventive measures mentioned, hopefully only a few cases will require buckling surgery. Injectable and degradable materials are eligible candidates for use in scleral buckling. Polymers that are liquid at room temperature, but become solid at body temperature, can be injected into the globe overlying a retinal break with the guide of indirect ophthalmoscopy. These liquids can be made of biodegradable materials to minimize long-term anatomical alterations, such as corneal aberrations.^17^

Injections are simple, do not have a learning curve, do not need much anesthesia, are fast and cheap, and can be performed in outpatient settings. They are obviously much more acceptable to patients than surgery. They are much more efficient in terms of time and money, and are much less disturbing to the patient, care providers and the eye. This is why I consider them to be the future of vitreoretinal surgery.

## References

[1] Fujii GY, De Juan E, Humayun MS, Pieramici DJ, Chang TS, Awh C (2002). A new 25-gauge instrument system for transconjunctival sutureless vitrectomy surgery. Ophthalmology.

[2] Eckardt C (2005). Transconjunctival sutureless 23-gauge vitrectomy. Retina.

[3] Holekamp NM (2010). The vitreous gel: more than meets the eye. Am J Ophthalmol.

[4] Holekamp NM, Shui YB, Beebe DC (2005). Vitrectomy surgery increases oxygen exposure to the lens: a possible mechanism for nuclear cataract formation. Am J Ophthalmol.

[5] Ackerman MJ (2009). Personalized medicine. J Med Pract Manage.

[6] Wassélius J, Johansson K, Håkansson K, Abrahamson M, Ehinger B (2005). Cystatin C uptake in the eye. Graefes Arch Clin Exp Ophthalmol.

[7] Sebag J (1998). Pharmacologic vitreolysis. Retina.

[8] Zhi-Liang W, Wo-Dong S, Min L, Xiao-Ping B, Jin J (2009). Pharmacologic vitreolysis with plasmin and hyaluronidase in diabetic rats. Retina.

[9] Rizzo S, Pellegrini G, Benocci F, Belting C, Baicchi U, Vispi M (2006). Autologous plasmin for pharmacologic vitreolysis prepared 1 hour before surgery. Retina.

[10] Wang ZL, Zhang X, Xu X, Sun XD, Wang F (2005). PVD following plasmin but not hyaluronidase: implications for combination pharmacologic vitreolysis therapy. Retina.

[11] Murakami T, Takagi H, Ohashi H, Kita M, Nishiwaki H, Miyamoto K (2007). Role of posterior vitreous detachment induced by intravitreal tissue plasminogen activator in macular edema with central retinal vein occlusion. Retina.

[12] Yamamoto S, Sugita S, Sugamoto Y, Shimizu N, Morio T, Mochizuki M (2008). Quantitative PCR for the detection of genomic DNA of Epstein-Barr virus in ocular fluids of patients with uveitis. Jpn J Ophthalmol.

[13] Takano A, Hirata A, Ogasawara K, Sagara N, Inomata Y, Kawaji T (2006). Posterior vitreous detachment induced by nattokinase (subtilisin NAT): a novel enzyme for pharmacologic vitreolysis. Invest Ophthalmol Vis Sci.

[14] Wang F, Wang Z, Sun X, Wang F, Xu X, Zhang X (2004). Safety and efficacy of dispase and plasmin in pharmacologic vitreolysis. Invest Ophthalmol Vis Sci.

[15] Stalmans P, Delaey C, de Smet MD, van Dijkman E, Pakola S (2010). Intravitreal injection of microplasmin for treatment of vitreomacular adhesion: results of a prospective, randomized, sham-controlled phase II trial (the MIVI-IIT trial). Retina.

[16] Hermel M, Dailey W, Hartzer MK (2010). Efficacy of plasmin, microplasmin, and streptokinase-plasmin complex for the in vitro degradation of fibronectin and laminin- implications for vitreoretinal surgery. Curr Eye Res.

[17] Okamoto F, Yamane N, Okamoto C, Hiraoka T, Oshika T (2008). Changes in higher-order aberrations after scleral buckling surgery for rhegmatogenous retinal detachment. Ophthalmology.
